# An examination of Ireland’s sugar sweetened beverage tax (sugar tax) in practice

**DOI:** 10.1093/pubmed/fdad097

**Published:** 2023-06-20

**Authors:** Frank Houghton, Jennifer Moran Stritch, Loveth Nwanze

**Affiliations:** Technological University of the Shannon, Social Sciences ConneXions, Limerick, V94 EC5T, Ireland; Technological University of the Shannon, Social Sciences ConneXions, Limerick, V94 EC5T, Ireland; School of Medicine, University of Limerick, Limerick, V94 T9PX, Ireland

**Keywords:** Ireland, pass-through rate, price, SSBT, sugar sweetened beverage tax, sugar tax

## Abstract

**Background:**

In the face of rising obesity levels, Ireland introduced a sugar sweetened beverage tax (SSBT) in 2018, the scope of which was extended in 2019. To date, there is a dearth of research on the actual impact of the SSBT on the pricing.

**Method:**

This study involved an examination of the relative cost of leading brand full-sugar and sugar-free carbonated soft drinks in a convenience sample of 14 different Irish supermarkets. In light of manufacturers’ reformulation of certain brands (7UP, Sprite and Fanta), information was collected on the relative in-store pricing of three brands (Coca Cola, Pepsi and Club).

**Results:**

In-store comparisons of equivalent size and unit number indicate that, in ~60% of cases, the full-sugar and sugar-free versions of the same drink are being offered at the same price. Even when full-sugar versions of these brands were more expensive than the sugar-free alternatives, the price differential was sometimes less than the SSBT rate.

**Conclusions:**

The pass-through rate of the SSBT to consumers is sub-optimal. Future policy and research suggestions are outlined.

## Background

Ireland, like many countries globally, is experiencing unprecedented high rates of obesity among its population.^1^ The World Health Organization (WHO) has referred to this global epidemic as ‘Globesity’.^2^ Obesity is linked to diabetes, cancer and cardiovascular disease,^3^ while high sugar intake is linked to tooth decay and gum disease.^4^^,^^5^ In response to the high proportion of its population who are overweight or obese, Ireland introduced the sugar sweetened beverage tax (SSBT), which is often referred to simply as the sugar tax.^6^^,^^7^

The Irish government introduced the SSBT in the face of significant opposition from industry.^8–10^ Although the Irish government have received many accolades for introducing the SSB tax, it has been noted that this tax was only introduced after an end to EU agricultural subsidies effectively terminated the Irish sugar beet industry,^11^ and at a time of declining full-sugar soft drink consumption.^12^ Although the revenue accruing from the SSBT has fallen short of expectations,^13^ the Irish government has still been condemned for not ringfencing taxes collected through the SSB tax for spending on health and combating obesity.^14^

Ireland’s legislation imposes a tax of €16.26 per hectolitre for certain beverages containing at least 5 g of sugar per 100 ml, but <8 g. This would equate to a tax of 5 cents on standard 330-ml cans, 8 cents on a 500-ml bottle and 16 cent on a litre bottle. For beverages containing sugar ≥8 g per 100 ml, the tax is €24.39 per hectolitre. This is 24 cents per litre, 12 cents for a 500-ml bottle and 8 cents on a 330-ml can.^15^ Ireland’s SSBT was introduced on 1 May 2018. It was subsequently amended on 1 January 2019 via the Finance Act 2018, which extended its scope to include specific plant protein drinks as well as drinks containing milk fats.^16^

The sugar tax is a classic example of a so-called sin tax, a tax applied to a product or service, which is often viewed as harmful to society. Routine examples include tobacco, alcohol and gambling. Sugar taxes are increasingly common, with >50 countries worldwide having introduced such taxes to date.^17^ Opposition to such interventions are often based on grounds such as civil liberties, the regressive nature of such VAT-based levies and concerns over targeting of specific industries in an internal free market such as that operated by the EU.^18^ It has also been observed that governments may simply be profiting from the additional revenue in their collection of many sin taxes.^18^ However, such arguments have been found to be misplaced, unfounded or insubstantial.^19^^,^^20^ Although regressive by nature, the SSBT retains potential advantages in promoting health equity. The prevalence of childhood obesity is significantly higher in children from lower-income backgrounds.^21^^,^^22^ Therefore, the SSBT has the potential to disproportionately promote the health of children from such backgrounds, offsetting the regressive impacts of this form of taxation.^23^ The WHO supports the introduction of such targeted taxes ^24^^,^^25^. It should be noted that, although it has been suggested that soft drink preferences are relatively inelastic,^26^ substantial literature exists to support such interventions.^20^^,^^27–33^

It is also possible that the introduction of a sugar tax has more of a psychological than practical impact on customers. Similar to ‘traffic-light’-style warnings on food,^34^ knowledge of the tax could act as a ‘red flag’ to consumers. However, Claudy *et al*. have noted an excessive focus in the academic literature around SSB on demand-side issues, ignoring supply-side strategies such as reformulation and pass-through rates.^20^ Andreyeva *et al*. have suggested that, on average, 82% of SSBTs are passed on to consumers.^33^

This study sought to examine how the SSBT impacted the consumer prices across a range of products and sizes in typical supermarkets in Ireland.

## Method

A convenience study of carbonated soft drinks in 14 different leading chain supermarkets in a provincial Irish city was undertaken in Spring 2022. This study focussed on the most popular traditional carbonated soft drinks, given their general appeal. As such high energy and high caffeine carbonated soft drinks were excluded. Initially, this examination focussed on the following leading soft drink brands: Coca Cola, Pepsi, 7UP, Sprite, Club and Fanta. However, the Fanta, 7Up and Sprite brands are all now below the 5 g of sugar per 100 ml (4.5, 4.7 and 4.5 g, respectively) and as such were excluded from subsequent analysis. Such changes in formulation have become increasingly common in recent years as manufacturers have sought to both assuage health concerns and avoid SSBT.^35–42^ This study examined the prices of full-sugar Coca Cola, Pepsi and Club drinks (which had 10.6, 11 and 12–13 g per 100 ml, respectively) versus their sugar-free equivalents.

## Results

Prices on 452 full-sugar and sugar-free Coca Cola, Pepsi and Club drinks were recorded across the 14 supermarkets. There was a substantial variation in the size of the cans and bottles examined. Eight different sizes, ranging from 150-ml cans to 2-l bottles were observed. There was a similarly high level of variation in the sizes of the multipacks for these products. In addition to single units, these items were available in 9 different multipack options ranging from 2 to 24 packs.

Tables 1 and 2 detail the results of price comparisons of full-sugar and sugar-free brands within the 14 supermarkets by brand, container size and multipack status. As can be seen from Table 1, 12 supermarkets sold both single 330-ml cans of Coca Cola and Diet Coke. In 42% (*n* = 5) of supermarkets, both cans were the same price. In 58% (seven) of cases, the full-sugar can was more expensive. In these seven cases, the price differential varied from 5 to 20 cents more expensive. Although the full-sugar coca cola was more expensive than Diet Coke in seven supermarkets, it is important to note that the sugar tax on such a can is 8 cents, not 5 cents.

**Table 1:** Comparison of single and multiple full-sugar & sugar-free cans

Brand and type	Multiple <330-ml cans	Single 330-ml can	Multiple 330-ml containers ^a^
Coca Cola versus Diet Coke	*N* = 1 LP = 0% (0) SP = 100% (1) HP = 0% (0)	*N* = 12 LP = 0% (0) SP = 42% (5) HP = 58% (7) (RD = +0.05−+0.20)	*N* = 6 LP = 0% (0) SP = 17% (1) HP = 83% (5) (RD per 330 ml = +0.06−+0.125)
Coca Cola versus Coke Zero	—	*N* = 12 LP = 0% (0) SP = 50% (6) HP = 50% (6) (RD per 330 ml = +0.10−+0.20)	*N* = 2 LP = 0% (0) SP = 0% (0) HP = 100% (2) (RD per 330 ml = +0.10)
Coca Cola versus Diet Coke No Caffeine	—	*N* = 2 LP = 0% (0) SP = 100% (2) HP = 0% (0)	—
Pepsi versus Pepsi Max	—	*N* = 6 LP = 0% (0) SP = 33% (2) HP = 66% (4) (RD per 330 ml = +0.05−+0.20)	*N* = 2 LP = 0% (0) SP = 100% (2) HP = 0% (0)
Pepsi versus Diet Pepsi	—	*N* = 5 LP = 0% (0) SP = 100% (5) HP = 0% (0)	*N* = 2 LP = 0% (0) SP = 100% (2) HP = 0% (0)
Club Orange versus Club Zero Orange	—	*N* = 8 LP = 0% (0) SP = 63% (5) HP = 37% (3) (RD per 330 ml = +0.20−+0.35)	*N* = 7 LP = 0% (0) SP = 43% (3) HP = 57% (4) (RD per 330 ml = +0.08)
Club Lemon versus Club Zero Lemon	—	—	—
Club Rock Shandy versus Club Zero Rock Shandy	—	—	—

LP, percentage and number of instances when the full-sugar drink was a lower price than the sugar-free equivalent; SP, percentage and number of instances when the full-sugar drink was the same price as the sugar-free equivalent; HP, percentage and number of instances when the full-sugar drink was a higher price than the sugar-free equivalent; RD, range of different prices observed in Euros.

^a^Multipacks were available in 9 different multipack options ranging from 2 to 24 packs.

^b^Instances where the sugar-free drink was more expensive than the full-sugar drink.

**Table 2:** Comparison of single and multiple full-sugar and sugar-free bottles

Brand and type	Single 500-ml bottle	Multiple 500-ml bottles	Single bottle, 1–2 l	Multiple bottles, 1–2 l
Coca Cola versus Diet Coke	*N* = 13 LP = 0% (0) SP = 46% (6) HP = 54% (7) (RD = +0.04−+0.20)	*N* = 2 LP = 0% (0) SP = 0% (0) HP = 100% (2) (RD per 500 ml = +0.10)	*N* = 14 LP = 0% (0) SP = 71% (10) HP = 29% (4) (RD per litre = +0.25−+0.34)	—
Coca Cola versus Coke Zero	*N* = 13 LP = 0% (0) SP = 46% (6) HP = 54% (7) (RD = +0.04−+0.20)	—	*N* = 7 LP = 0% (0) SP = 100% (7) HP = 0% (0) (RD per petre = 0)	*N* = 6 LP = 0% (0) SP = 50% (3) HP = 50% (3) (RD per 330 ml = +0.03−+0.44)
Coca Cola versus Diet Coke No Caffeine	—	—	*N* = 3 LP = 0% (0) SP = 67% (2) HP = 33% (1) (RD per 330 ml = +0.03)	—
Pepsi versus Pepsi Max	*N* = 10 LP = 20% (2)^*^ SP = 40% (4) HP = 40% (4) (RD per 500 ml = −0.15 to +0.55)	—	*N* = 6 LP = 17% (1)^*^ SP = 33% (2) HP = 50% (3) (RD per litre = −0.25 to +0.30)	—
Pepsi versus Diet Pepsi	*N* = 5 LP = 0% (0) SP = 40% (2) HP = 60% (3) (RD per 500 ml = +0.10−+0.20)	*N* = 5 LP = 0% (0) SP = 80% (4) HP = 20% (1) (RD per 500 ml = +0.15)	*N* = 5 LP = 60% (3)^*^ SP = 40% (2) HP = 0% (0) (RD per litre= −0.25)	—
Club Orange versus Club Zero Orange	*N* = 11 LP = 0% (0) SP = 64% (7) HP = 36% (4) (RD per 500 ml = +0.04−+0.15)	—	*N* = 9 LP = 0% (0) SP = 67% (6) HP = 33% (3) (RD per litre = +0.09−+0.26)	—
Club Lemon versus Club Zero Lemon	—	—	*N* = 6 LP = 0% (0) SP = 50% (3) HP = 50% (3) (RD per litre = +0.07−+0.15)	—
Club Rock Shandy versus Club Zero Rock Shandy	—	*N* = 10 LP = 0% (0) SP = 90% (9) HP = 10% (1) (RD per litre = +0.15)	-	—

LP, percentage and number of instances when the full-sugar drink was a lower price than the sugar-free equivalent; SP, percentage and number of instances when the full-sugar drink was the same price as the sugar-free equivalent; HP, percentage and number of instances when the full-sugar drink was a higher price than the sugar-free equivalent; RD, range of different prices observed in Euros.

^a^Instances where the sugar-free drink was more expensive than the full-sugar drink.

It is particularly notable in Table 2 that the larger size bottles of full-sugar drinks, sold individually or in bundles, were routinely sold at the same price as the equivalent sugar-free bottles. Even when a higher price is charged, it is notable here again that there are multiple examples of the price differential being lower than expected as a result of the sugar tax. One finding that was particularly alarming was the occasional instances where the diet version of the soft drink examined was more expensive than the full-sugar equivalent.

It should also be noted in Tables 1 and 2 that there are also multiple examples of the full-sugar price differential being substantially higher than that resulting purely from the sugar tax. Overall, in 107 instances, the same price was charged for full-sugar and sugar-free Coke, Pepsi and Club brand drinks. In 77 cases, higher prices for the full-sugar version of these brands was more expensive than the sugar-free version, although the sums charged ranged above and below than anticipated purely by the SSBT. In six cases, the prices of the sugar-free drinks were more expensive than their full-sugar equivalents.

The analysis above is based on specific comparisons of prices. It should be noted that five of the supermarkets examined also offered eight ‘bargain bucket’ style offers with multiple bottles and cans of the brands examined available for a set price. Thus, overall, where exact comparisons could be made, in approximately, three-fifths of cases (115/198; 58%) the full-sugar and the sugar-free versions were being sold at the same price.

The total number of specific comparisons made was less than anticipated. The significant level of variation in the product size and number reduced the number of within-store direct product comparisons that were possible. Thus, for example, in some supermarkets, multipacks of 10 and 20 cans of full-sugar drinks were available alongside 12- and 24-can multipacks of same brand sugar-free drinks; equal-size equivalent packs not being stocked. This form of differentiation making any clear assessment of potential price differences particularly difficult.

## Discussion

It is evident from this research that, despite the introduction of a SSBT in Ireland, customers are often not being charged extra for high-sugar drinks in supermarkets. The pass-through rates on the SSBT are substantially lower than expected in ~60% of cases where direct comparisons were possible. Such a lack of price differentiation clearly undermines the effectiveness of the SSBT in influencing consumer choices. One possible option may be to increase the SSBT rate to ensure outlets pass the tax on to consumers. A commitment by government to ring-fence taxes collected in this manner for expenditure on obesity-related health promotion, research and intervention would probably make such a move more politically acceptable.^43^ Given significant increases in inflation in recent months (Trading Economics, 2022),^44^ an increase in the SSBT may already be appropriate to maintain or enhance its impact.

From a public health perspective, the reformulation of the Fanta, 7UP and Sprite brands to reduce their sugar content to <5 g per 100 ml should be seen as a success. It is, though, an issue of concern that the other brands assessed in this study (Coca Cola, Pepsi and Club) were not only subject to the SSBT but were also well into the high-sugar bracket (≥8 g per 100 ml). Further work with the industries behind these brands is urgently required to convince them to reduce their sugar levels. Research should be conducted with the major chain supermarkets to explore their decision in many cases to absorb the higher tax rate on high-sugar drinks rather than passing such costs on to consumers.

This research should also be extended to include high-energy and caffeine drinks, as well as products such as specific plant protein drinks, and drinks containing milk fats, as included in the expanded SSBT legislation in 2019. Further research should also examine similar pricing structures of drinks subject to the SSBT in other settings, for example, in bars, pubs, restaurants and hotels. Finally, it is suggested that the differentiation in the quantities sold in multipacks is explored. It is possible that slightly smaller multipacks of full-sugar drinks are being sold at an equal or very similar price to multipacks.

## Data Availability

The data underlying this article will be shared on reasonable request to the corresponding author.

## Notes

**F. Houghton**, Director of Social Sciences

**J. M. Stritch**, Principal Investigator

**L. Nwanze**, Master, Postgraduate Student
